# Case report: Long term follow-up of a large unilateral epididymal cyst in a stallion used for teaching: Is this condition associated with infertility?

**DOI:** 10.3389/fvets.2023.1145742

**Published:** 2023-03-29

**Authors:** Gabriela Fernandes Silva, Raquel Cunha, Fátima Carvalho, Mário Ribeiro, António Rocha, Irina Amorim, Tiago Guimarães

**Affiliations:** ^1^School of Medicine and Biomedical Sciences (ICBAS), University of Porto (UP), Porto, Portugal; ^2^Center for the Study of Animal Sciences (CECA), ICETA, University of Porto, Campus Agrário de Vairão, Vairão, Portugal; ^3^Institute of Molecular Pathology and Immunology, University of Porto (IPATIMUP), Porto, Portugal; ^4^Institute for Research and Innovation in Health (i3S), University of Porto (UP), Porto, Portugal

**Keywords:** equine, epididymis, cyst, semen, spermatocele, sperm granuloma

## Abstract

A 30-year-old Lusitano stallion presented with an enlarged right epididymis. The ultrasound scan revealed a cyst-like formation and the histopathological examination was compatible with epididymal cyst located at the body/tail transition, epididymal spermatocele and sperm granuloma and epididymitis. However, these conditions did not seem to affect the animal's reproductive performance, nor did the semen parameters analyzed over the 8 years after the diagnosis show significant changes. Nevertheless, since the ejaculate contains mostly sperm cells from the tail of the epididymis, where fertile spermatozoa are stored until ejaculated, a deep knowledge of the different conditions that can affect this organ is of the utmost importance.

## Introduction

Most of the available literature regarding male genital pathology focuses on the testes and abnormalities of spermatogenesis. Routine pathologic examination of the epididymis is often neglected and epididymal pathologies are often undervalued.

The epididymis is crucial for the concentration, maturation, transport and storage of sperm (1, 2). Spermatozoa are produced in the seminiferous epithelium, leave the testis, and enter the efferent ductules that lead them to the epididymis. In the epididymis, they are successively transported to the head and body, where they mature through sequential exposure to different fluids and in the tail of the epididymis sperm are stored until ejaculation (3). Accumulated secretions can cause enlargement of the epididymis, forming cystic structures that can occur in all regions, but which are more commonly found in the head and tail. Clinically, epididymal cysts are palpated as extratesticular masses, usually smooth, round, and characteristically located within the epididymis, that can be incidentally detected during physical examination or ultrasound examination of the scrotal contents. Of the three portions of the epididymis, the tail is the most easily palpable, making it easier to detect a cystic structure in this location, while in the others it may go by unnoticed (4).

Epididymal cysts are a collection of fluid in a single (unilocular) or multiple (multilocular) cavities as a result of the dilation of the efferent epididymal tubules, due to partial or complete tubular obstruction. These epididymal cysts are usually translucent, as they contain clear fluid, but a few, due to the presence of spermatozoa, may present a turbid appearance. These lesions are typically unilateral and benign in nature. The terms “epididymal cysts” and “spermatoceles” have been used interchangeably on several occasions to describe the same entity (5). However, the final diagnosis and accurate classification of epididymal cysts is based on the precise location and histological features of the cyst wall and adjacent tissue (6).

Although rare in the stallion, epididymal cysts can occur in blind vestiges of the extratesticular rete tubules (blind efferent ductules) or mesonephric tubules (aberrant tubules), in the blind cranial portion of each mesophrenic duct (appendix of the epididymis), or in detached segments of the mesonephric tubules (paradidymis) (7, 8).

In this report, we describe the clinical and microscopic findings associated with an enlarged right epididymis of a 30-year-old Lusitano stallion presenting with epididymal cysts. These lesions were confirmed and monitored through consecutive ultrasound scans since 2011. Additionally, the respective reproductive data of this animal are analyzed and discussed. Based upon all the results, some inferences about the pathophysiology and histogenesis of this lesion are drawn.

## Case description

A 30-year-old Lusitano stallion, routinely used for breeding soundness evaluation and semen collection during the Theriogenology classes at ICBAS Veterinary School, was euthanized in October 2020 due to untreatable chronic health problems such as recurrent colics, several episodes of laminitis and an enlarged right epididymal tail that was first detected in 2011. Echographic examination revealed an epididymal cyst-like lesion that was monitored from 2011 until 2020, through several ultrasound scans. Therefore, testes and epididymides were collected, fixed in 10% neutral buffered formalin, and sent for histopathological evaluation at ICBAS-UP Veterinary Pathology Laboratory.

**Figure 1:**
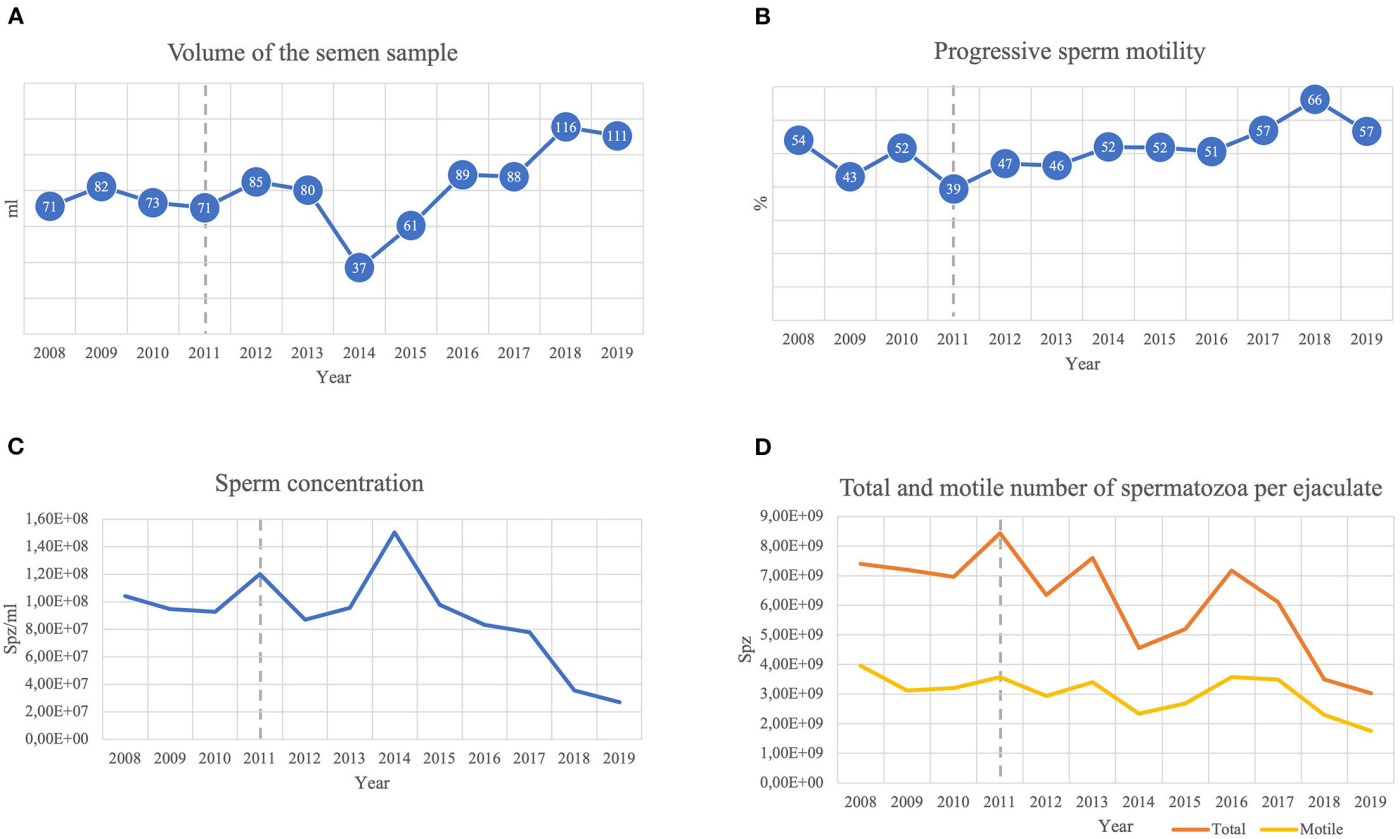
Stallion reproductive parameters collected during 2008–2019: **(A)** total semen sample volume collected; **(B)** progressive sperm motility; **(C)** sperm concentration; **(D)** total and motile number of spermatozoa per ejaculate. An abrupt decrease in the volume of the semen sample associated with a peak in total sperm concentration was noted in 2014, although the numbers of total and motile spermatozoa present were reduced. In 2018 and 2019, the highest values of semen volume were registered, accompanied by high values of progressive motility. Nevertheless, sperm concentration decreased from 2015 to 2019, presenting a decrease in the number of total and motile spermatozoa that reached minimal values in 2019. - - Lesion's clinical diagnosis.

Throughout these years, no signs of inflammation other than enlargement of the right epididymis could be felt during palpation of the external genitalia, and the stallion did not present any evidence of pain or discomfort during physical examination. The stallion displayed excellent libido, and semen could be easily collected, even at the peak of winter, with an artificial vagina and a phantom as a mount, without the need for a live mare for teasing. Semen evaluation was also frequently performed in classes and results of the reproductive parameters collected from 2008 to 2019 are depicted in Figure 1. Although the stallion was seldom used for breeding, fertility results were considered good: four foals were born after a single artificial insemination (AI) with fresh semen, and 7 embryos were collected from donor mares, the last one in 2018, after a single AI with fresh semen. Sperm morphology assessed on “wet mounts” using phase-contrast microscopy (×1000) averaged 70% of normal sperm. A small trial of refrigeration (*n* = 3) suggested poor suitability for refrigeration, with a motility of fresh semen of 46%, decreasing to 10% in 24 hours. At the time of the clinical diagnosis, i.e., 2011, there was a slight decrease in the volume of semen collected, concomitant with an increase in its concentration. Furthermore, at this time, the lowest value of progressive sperm motility was registered.

**Figure 2:**
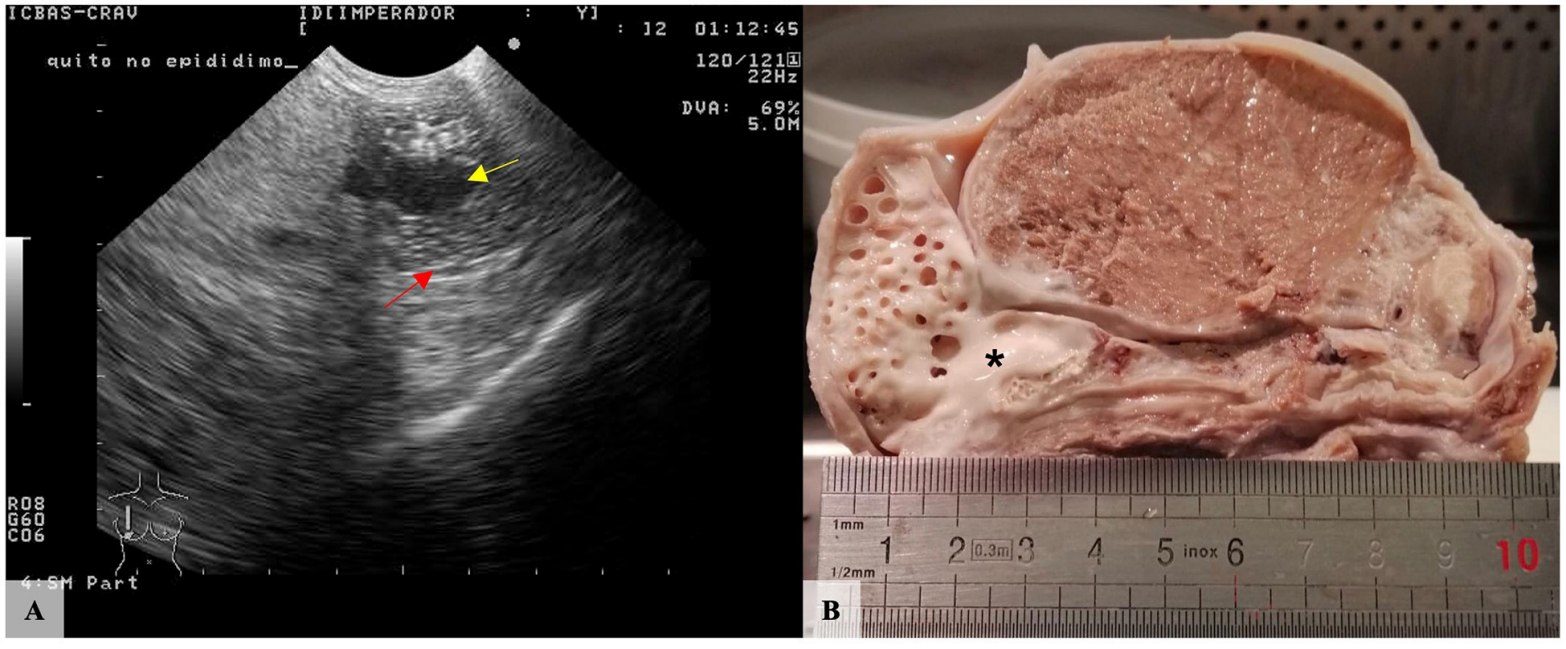
Purebred Lusitano stallion, right testis. **(A)** Ultrasound showed a well-circumscribed anechoic cystic structure (yellow arrow) in the epididymis. Red arrow: Epididymal wall. **(B)** Macroscopically, the largest cystic lesion (^*^) is filled with whitish and semen-like material located in the terminal portion of the body of the epididymis. Several smaller cavities were also identified in the tail of the epididymis.

Also in the context of classes, consecutive testicular, epididymal and spermatic cord ultrasounds were performed at least four times per year since 2005, using an Aloka ultrasound scanning model Prosound, mostly with a 5.0 MHz sector transducer. No deviations from the expected ultrasound images were seen in the epididymis. However, in 2011, a cyst-like structure was easily detected in the tail of the right epididymis, apparently measuring 1.8 cm in its largest dimension (Figure 2A). By this time, the parenchyma of both testes exhibited a uniformly echogenic homogenous image. Additionally, no alterations of the bulbourethral glands, prostate, vesicular glands or ampullae of the ductus deferens were ever noticed.

In 2020, by the time of death, no relevant alterations were identified during general physical examination apart from the epididymal alteration that measured ~2.6 cm in its largest dimension. The excised right testis measured 10.0 × 5.5 × 5.0 cm in its largest dimensions, and, after longitudinal section, multiple cavities filled with white, opaque and semi-solid content were observed in the epididymis. The most prominent and largest cavity was located between the caudal third of the body of the epididymis and the beginning of the tail and measured about 1.0 cm in diameter. Additionally, several well-defined cavities with similar content were observed, replacing the entire tail of the organ and measuring between 0.2 and 0.7 cm in diameter (Figure 2B). In contrast, the left testis measured 7.5 × 5.5 × 4.0 cm in its largest dimensions and no relevant macroscopic alterations were observed at cross-section. Multiple sections of both organs were cut, paraffin-embedded, and consecutive 2μm-thick sections were routinely processed for microscopic evaluation.

**Figure 3:**
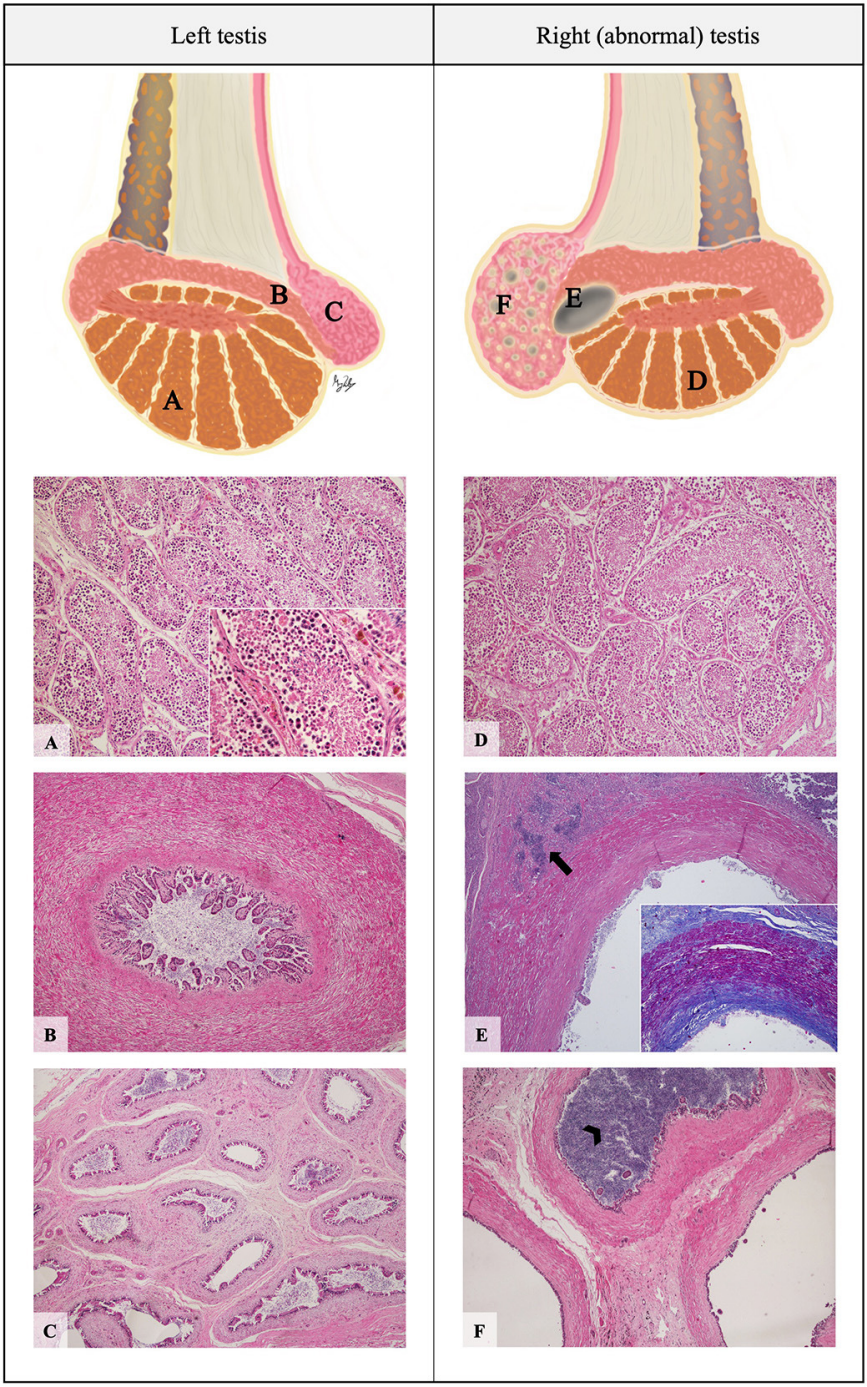
Schematic representation and histopathological photomicrographic of the stallion's testes. Left testis: parenchyma **(A)**, epididymal body **(B)** and epididymal tail **(C)**. Right testis: parenchyma **(D)**, epididymal cyst **(E)** and epididymal tail **(F)**. **(A, D)** Seminiferous ducts surrounded by germinal epithelium containing a large amount of exfoliated germ cells and round bodies in the lumen (H&E, 100x); Inset: Presence of spermatocytes, spermatids, and some spermatozoa in the lumen of the ducts (H&E, 400x). **(B)** Normal epididymal body (H&E, 40x). **(E)** Dilated cavity containing spermatozoa in the lumen, associated with foci of granulomatous reaction- spermatic granuloma (arrow) (H&E, 40x); Inset: The lesion is circumscribed by fibrosis and a dense muscular layer, evidenced by Masson's Trichrome histochemical staining (40x). **(F)** Enlarged ducts in the tail of the epididymis, compared to those of the normal contralateral testis; Note the sperm stasis in some ducts which are filled with sperm (arrowhead) (H&E, 40x).

**Figure 4:**
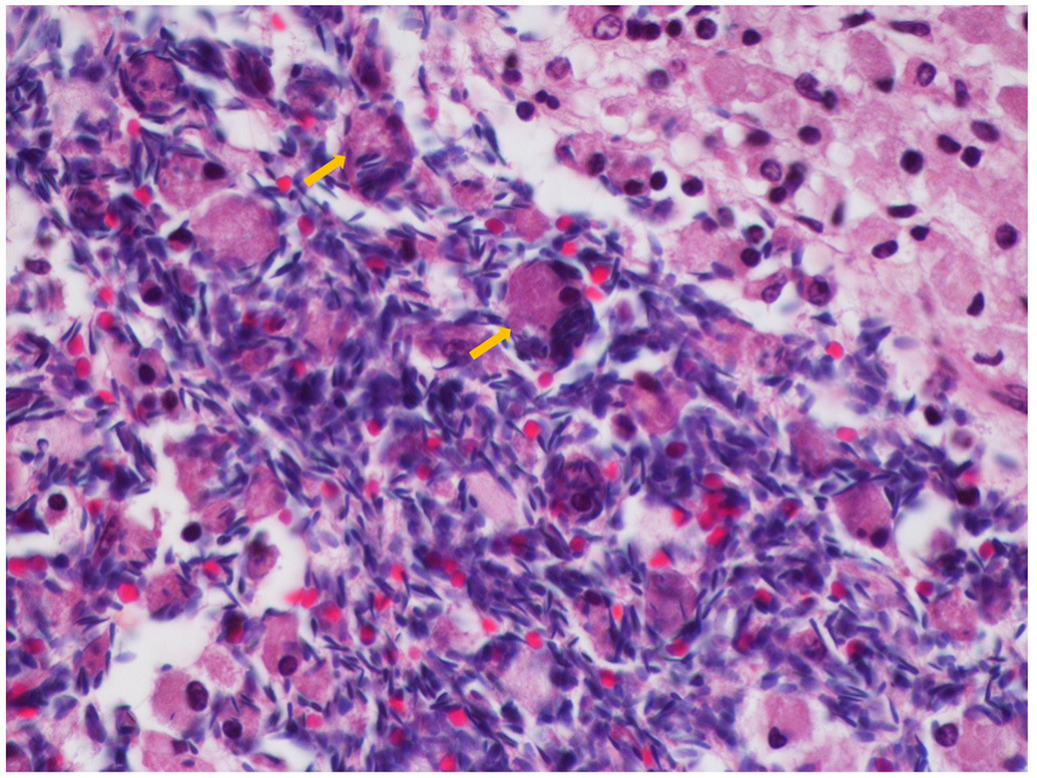
Foreign body inflammatory reaction in response to sperm extravasation, consisting of numerous multinucleated giant cells (arrows) (H&E, 600x).

Histological evaluation showed a multilocular cystic lesion extending from the terminal portion of the body to the tail of the epididymis. The lesion consisted of several cavities of variable size with walls lined by a multifocally hyperplastic and low columnar ciliated epithelium, externally circumscribed by well-organized smooth muscle bundles that stained accordingly with Masson Trichrome (Figure 3). Abundant sperm was present in the lumen of these cystic structures. In the adjacent tissue, several foci of foreign body inflammatory reaction centered in free sperm were observed, composed of numerous macrophages exhibiting intense phagocytic activity, multinucleated giant cells and plasma cells (Figure 4). Based on macro- and microscopic findings, the lesions were diagnosed as spermatocele, as the cystic structure was filled with sperm, and sperm granuloma due to local immune foreign body (sperm cells) inflammatory reaction, and epididymitis.

## Discussion

Testicular and epididymal cystic lesions have been reported in several species including canine (9), feline (10), equine (6), bovine (11), ovine (12), porcine (13), caprine (14), and humans (15). Epididymal cysts are mainly of congenital origin, but traumatic or inflammatory causes must be considered (16). Some reports of equine testicular cysts have also been associated with teratomas (6). In the described case, the precise cause of this pathological condition could not be determined.

Ultrasonography is considered the method of choice for identifying congenital or acquired epididymal cysts, since it is easily performed with minimal animal restraint or discomfort and provides highly sensitive information and accurate diagnosis (17, 18). However, in this case, ultrasonography was not able to determine the precise anatomical location of the largest cyst, nor the detection of the other smaller cystic lesions. Indeed, the resolution of the ultrasound equipment, equipped with a 5 MHz probe, was not suitable for the accurate detection of the smaller lesions. Nevertheless, Pozor (18) reported that most equine epididymal cysts detected by ultrasound scanning occur in the head, contrary to our findings. This highlights the importance of careful screening of all epididymal sections.

Macroscopically, cysts in the epididymis are characterized by an increase in the size and asymmetry of the epididymis in comparison with the contralateral organ (2), findings consistent with the current case. However, the final diagnosis and classification of these types of lesions rely on the precise location and histological features of the cyst wall and adjacent tissue (6).

The different cell populations of the epididymal epithelium are responsible for its wide range of functions (absorption, secretion, and exchange of ions), creating the intraluminal microenvironment that enables the transformation of immature testicular spermatozoa into mature sperm (19). The epididymis of most mammals is a single, highly convoluted duct, lined by a complex pseudostratified epithelium composed of two main cell types (principal and basal cells) as well as other types of accessory cells (apical cells, clear cells, and intraepithelial leukocytes) varying in number and appearance across the various segments (20). The thickness of the intertubular connective tissue and muscle layer increases from the head to the tail region of the epididymis. Furthermore, in the tail of the epididymis, the epithelium is comparatively thinner and the surrounding smooth muscle more abundant (21). Histologically, epididymal cystic structures are lined by epithelium similar to that of the epididymis and usually contain a clear luminal fluid (2, 8).

The difference between epididymal cysts and spermatoceles is not clear. In clinical terms, they are indistinguishable on palpation, produce a similar ultrasound image and the most viable method of differentiating them is through aspiration of their contents, a process that is not always clinically indicated, especially if they are asymptomatic (22, 23). Epididymal cysts are filled with serous fluid of probable lymphatic origin and spermatoceles are usually due to obstruction of the efferent ductal system, leading to a cystic dilation filled with spermatozoa (24). Thus, it is suggested that spermatoceles are a form of epididymal cyst with a different fluid composition. However, as they have similar clinical relevance being treated only in symptomatic cases, they are often described in the literature as the same entity and both terms have been used interchangeably (23).

In this stallion, the histological features observed, namely the lower columnar epithelium surrounded by abundant muscle bundles, as well as the anatomical location of the lesion, support the diagnosis of an epididymal cystic lesion. As the cystic cavity was filled with fluid compatible with sperm, the lesion is defined as a spermatocele (15). This lesion presumably led to complete spermatic blockage or spermiostasis, characterized by the accumulation of an excessive number of sperm, which can cause prominent dilation of the epididymis (25). It is, however, interesting to note that, contrary to this case, spermiostasis occurs often in the head of the epididymis and is usually caused by birth defects such as segmental aplasia and aberrant or blind efferent ductules (26, 27).

This animal was clinically diagnosed with epididymitis due to the enlargement of the right epididymal tail and the cyst-like structure detected by ultrasound. In the stallion, epididymitis is usually of infectious origin and is relatively uncommon, often involving uni- or bilateral disease of the epididymal tail, frequently with painful enlargement of the epididymis (28). In stallions, migrant strongyles larvae are considered a possible cause of epididymitis and spermatic granuloma, and rare cases of bacterial epididymitis are associated with *Streptococcus equi* subsp. *zooepidemicus* (2). However, this animal showed no signs of infectious disease, nor apparent inflammation of the accessory genital glands, injuries described to be associated with infectious epididymitis (2). Epididymitis can also occur due to congenital ductal anomalies, adenomyosis, trauma, and as a consequence of spermatic granulomas (2). In the current case, no signs of pain were noticed, the epididymal enlargement seemed to be related only to the cystic lesion, and the observed inflammatory condition was possibly a simple consequence of the cystic lesion. The spermatic granuloma observed in this animal is a common finding in stallions diagnosed with epididymal cysts, since sperm stasis inside the structure may cause the eventual destruction of the ductal epithelium by the spermatozoa, allowing them to escape into the adjacent tissues and induce a foreign body reaction and granuloma formation (26, 27). In turn, the chronic inflammation and fibrosis induced by this reaction can lead to the complication of ductal obstruction (2). Because of the disruption of the blood-testis barrier, anti-sperm antibodies may subsequently be formed. Anti-sperm antibodies are found in serum and in genital secretions, and their presence has been associated with infertility in stallions (2, 29).

Several authors report that epididymal cysts, specifically those located in the head, are associated with ejaculatory dysfunctions. However, they can be observed in stallions without any alteration of semen parameters (4, 28). In addition, obstruction of any portion of the ductal lumen, such as the epididymal cyst found in this stallion, can cause a marked reduction in sperm quality or azoospermia (25), preventing normal sperm maturation and decreasing motility values (30). Thus, the analysis of semen parameters is essential in order to detect some anomalies in the epididymis, as they may reflect epididymal performance (30). Nevertheless, in the present case, the stallion did not display any ejaculatory dysfunction, had an acceptable percentage of morphologically normal sperm cells, and, despite an apparently low suitability for refrigeration of the ejaculate, artificial insemination with fresh semen suggested a good fertilizing ability. Therefore, in the reported case, analysis of the semen parameters are not suggestive of abnormal epididymal performance. It should be noted, however, that these values represent the spermatic function of both testicles, and the normal testis may have counterbalanced the alterations directly caused by the lesions.

The semen concentration and the number of total and motile spermatozoa per ejaculate of this animal decreased with time. The cyst in the right epididymis of this stallion resulted in spermiostasis and ductal blockage. However, the gradual decrease of concentration and sperm cells in the ejaculate cannot be attributed to an aggravation of spermiostasis with any degree of certainty. It is more plausible that other factors, such as the frequency of semen collection by different operators (students and staff), as well as age, may have contributed to this drop.

## Conclusion

Fertility problems are important aspects in the reproductive management of the stallion, so an assessment of reproductive health, including analysis of sperm parameters, physical examination and ultrasound of the genitalia, are essential for the diagnosis of anomalies. Additionally, for an accurate diagnosis of testicular, ductal, and epididymal lesions, histopathology plays a crucial role in determining the origin, location, and extent of the lesions. Since the epididymal tail is an essential reservoir of semen with adequate fertilizing ability, a detailed knowledge of the different pathophysiological conditions that can affect this organ is of the utmost importance. In the presented case, histopathological examination was essential in order to establish the type of condition detected clinically. The spermatocele certainly had a negative impact on the storage capacity of the tail of the right epididymis but it did not unequivocally interfere with the ability of sperm cell maturation and differentiation. Other possible deleterious effects on semen parameters could not be ascertained.

## Data availability statement

The original contributions presented in the study are included in the article/supplementary material, further inquiries can be directed to the corresponding author.

## Ethics statement

The studies involving animals were reviewed and approved by the local Ethics Committee of ICBAS-UP (Porto, Portugal), Animal Welfare Body (ORBEA), project no. 339/2019/ORBEA. Written informed consent from the owners for the participation of their animals in this study was not required in accordance with the national legislation and the institutional requirements. Written informed consent was obtained from the participant/patient(s) for the publication of this case report.

## Author contributions

GFS and IA wrote the manuscript. TG, AR, and RC carried out the clinical evaluation and clinical care and critically revised the manuscript for important intellectual content. FC carried out the HE staining. MR contributed to the formatting of images and schemes. All authors read and approved the final manuscript.
